# Transcutaneous Tibial Nerve Stimulation for Overactive Bladder Symptoms in Parkinson's Disease: Results from a Phase II Randomized Control Trial (STRIPE)

**DOI:** 10.1002/mds.30186

**Published:** 2025-04-17

**Authors:** Matthew D. Smith, Gabriella E. Portlock, Anisha Cullen, Anahita Nodehi, Marcus J. Drake, Yoav Ben‐Shlomo, Emily J. Henderson

**Affiliations:** ^1^ Ageing and Movement Research Group Population Health Sciences, Bristol Medical School, University of Bristol Bristol United Kingdom; ^2^ Department of Neurology North Bristol NHS Trust Bristol United Kingdom; ^3^ Department of Surgery and Cancer Faculty of Medicine, Imperial College London United Kingdom; ^4^ Older People's Unit Royal United Hospitals NHS Foundation Trust Bath United Kingdom

**Keywords:** autonomic, bladder, neuromodulation, overactive, Parkinson's

## Abstract

**Background:**

Lower urinary tract symptoms (LUTS) are common Parkinson's disease (PD), causing great impact.

**Objective:**

The goal was to undertake a phase II randomized control trial of transcutaneous tibial nerve stimulation (TTNS) delivered by Geko device for LUTS related to overactive bladder (OAB) in PD, an easy to use of the shelf solution.

**Methods:**

Participants were randomized to active/sham stimulation. Primary outcome measure was the International Consultation on Incontinence Questionnaire‐Overactive Bladder score (ICIQ‐OAB) at 12 weeks.

**Results:**

A total of 148 participants were allocated to active (73) and sham arms (75). No difference was seen between arms (coefficient, 0.48; 95% CI, −0.2 to 1.2; *P* = 0.17), although both active and sham showed improvements over baseline. Pain was the most common adverse event.

**Conclusion:**

No difference was seen between active and sham arms. Symptom improvements seen in both groups are consistent with a placebo effect, however, we cannot exclude a biological effect from the sham intervention. Although negative, this result should be taken only in context of Geko use rather TTNS in general. © 2025 The Author(s). *Movement Disorders* published by Wiley Periodicals LLC on behalf of International Parkinson and Movement Disorder Society.

Lower urinary tract symptoms (LUTS) are common in Parkinson's disease (PD)^1^ with predominant overactive bladder (OAB) symptoms including nocturia, urinary urgency, and urgency urinary incontinence. LUTS cause significant impact on quality of life^2^, ^3^ and contribute to morbidity and even mortality experienced by people with PD, resulting in falls and hospitalisation.^4^, ^5^

Various approaches have been investigated for LUTS in PD, including pharmacological and non‐pharmacological methods such as bladder training. The use of antimuscarnics can often be limited by side effects.^6^, ^7^, ^8^, ^9^ Minimally or non‐invasive neuromodulation approaches that include percutaneous or transcutaneous tibial nerve stimulation (TTNS) have increasingly been used for LUTS,^10^, ^11^ with the benefit of having a markedly lower side‐effect profile. Unfortunately, few of these studies have included a control comparator, so their true efficacy remains known. We undertook a randomized control trial (STRIPE—stimulation of the tibial nerve to improve incontinence in Parkinson's electronically) of TTNS using the Geko device in community ascertained people with PD as an easy‐to‐use solution.

## Methods

STRIPE was designed as a phase II randomized control trial (RCT) of TTNS against sham comparator, delivered by participants at home using the Geko device. STRIPE was undertaken between October 2021 and January 2024. It was approved by the Kings Cross and Camden Research Ethics‐Committee and the University of Bristol acted as sponsor. STRIPE was registered on the ISRCTN registry (1148495). The protocol for STRIPE has been published previously^12^ and is detailed in Supporting Information Section S1.

## Results

### Recruitment and Participant Flow

A total of 630 participants were assessed for trial eligibility, of which 166 (26.3%) attended a baseline assessment session. Eighteen (10.8%) potential participants were excluded because of clinically significant post void residual (n = 15, mean excluded volume 240 mL; range, 100–438 mL), inability to feel the device (n = 2), and inability to apply the device without prospect of external assistance (n = 1). The remaining 148 participants were enrolled and randomized (see CONSORT diagram Fig. S1).

### Baseline Characteristics

The mean age of participants was 69.7 years (range, 42–87; standard deviation [SD], 7.6 years) and median duration of PD 6 years, (interquartile range [IQR], 4–9 years). A total of 37.2% of enrolled participants were female. Baseline characteristics of participants are summarized for active and sham treatment arms in Table 1. The two arms were similar because of randomization, although the active arm had lower levodopa equivalent dose, International Consultation on Incontinence Questionnaire (ICIQ) female lower urinary tract symptoms (fLUTS) score, and nocturnal polyuria index.

**TABLE 1:** baseline characteristics of STRIPE cohort by treatment allocation

	Active	Sham	*P* value
	n = 73 (SD)	n = 75 (SD)	
Demographic and PD factors			
Age	69.0 (8.2)	70.3 (7.0)	0.32
% female	38.4%	36.0%	0.77
Duration of PD (y)	6.8 (3.7)	7.5 (5.1)	0.35
Levodopa equivalent dose (mg)	452 (178)	530 (255)	**0.04**.
MOCA score	27 (3.2)	27 (2.8)	0.70
Questionnaire scores totals			
ICIQ OAB	11.0 (2.0)	11.2 (2.0)	0.47
ICIQ OAB bother	22.7 (7.3)	21.9 (8.8)	0.55
ICIQ mLUTS	15.5 (5.8)	14.5 (5.1)	0.38
ICIQ mLUTS bother	46.3 (23.0)	38.1 (20.6)	0.08
ICIQ fLUTS	11.8 (3.9)	15.4 (5.4)	**0.006**
ICIQ fLUTS bother	43.6 (14.1)	50.9 (20.1)	0.13
ICIQ OAB QoL	61.5 (20.5)	67.1 (25.5)	0.14
SCOPA‐AUT	19.2 (7.9)	20.1 (8.7)	0.52
PHQ‐9	6.0 (4.4)	6.5 (5.3)	0.49
72‐h bladder diary and examination			
Day time frequency (per day)	7.5 (1.7)	7.3 (2.2)	0.53
Night time frequency (per day)	1.6 (1.0)	1.8 (1.3)	0.19
Urgency episodes (per day)	2.7 (2.2)	2.4 (2.4)	0.33
Incontinence episodes (per day)	0.6 (1.0)	0.6 (1.2)	0.99
Nocturnal polyuria index	34.5 (0.1)	39.3 (0.1)	**0.017**
Post void residual	41 (49)	36 (35)	0.46
% with orthostatic hypotension	39.1	38.6	0.95

*Note*: Bold *P*‐value denotes significant difference.

Abbreviations: STRIPE, stimulation of the tibial nerve to improve incontinence in Parkinson's electronically; SD, standard deviation; PD, Parkinson's disease; MoCA, Montreal Cognitive Assessment; ICIQ, International Consultation on Incontinence Questionnaire; OAB, Overactive Bladder; mLUTS, male lower urinary tract symptoms; fLUTS, female lower urinary tract symptoms; QoL, quality of life; SCOPA‐AUT, Scales for Outcomes in Parkinson's disease—Autonomic dysfunction; PHQ‐9, Patient Health Questionnaire 9.

### Compliance with Intervention and Trial Procedures

Following enrolment, seven participants withdrew and two lost to follow up. Six participants discontinued the intervention within the 12‐week period (5 active, 1 sham, *P* = 0.09), and 133 remained on treatment by the follow up consultation (95.7% of total). The difference in withdrawal between arms was consistent with chance (*P* = 0.73). Both participants lost to follow up were in the active arm. Within the active arm, 50 participants (68.4% of arm) were able to self‐administer and tolerate motor level stimulation (flexion or fanning of toes). The remaining 23 active participants (31.6%) used the device at highest tolerable level of sensory stimulation.

### Primary Analysis—Difference in ICIQ‐OAB Scores at Week 12

**FIG. 1:**
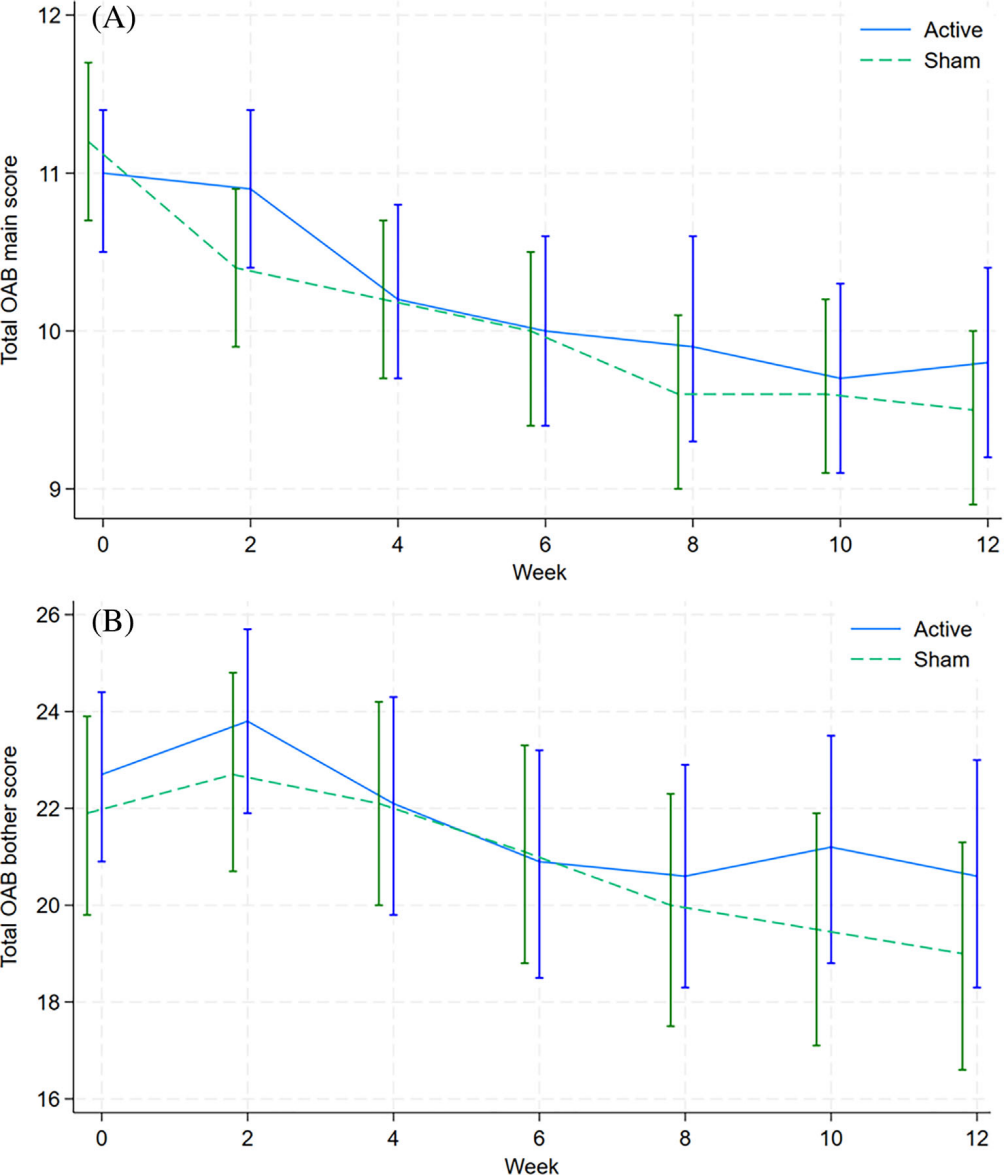
Line graph displaying trend for mean International Consultation on Incontinence Questionnaire‐Overactive Bladder (ICIQ‐OAB) score, including 95% confidence intervals over the course of the 12‐week intervention period for the intention to treat (ITT) population, for (**A**) main score and (**B**) bother score. [Color figure can be viewed at wileyonlinelibrary.com]

Mean total ICIQ‐OAB score at week 12 was not significantly different between arms using an adjusted model for both main score (regression coefficient, 0.31; 95% confidence interval [CI], −0.5 to 1.1; *P* = 0.17) or bother subscore (regression coefficient, 1.68; 95% CI, −1.6 to 5.0; *P* = 0.24)—see Figure 1 and Supplementary Table S1. Although there was a trend for the active intervention to have higher (and therefore, worse) ICIQ‐OAB scores, this difference was consistent with chance.

A further model using multiple imputation to account for missing data showed no significant difference in outcome (see Supplementary Section S2). No significant difference was demonstrated when controlling for unbalanced factors at baseline or stratification by sex or level of symptom severity (Supplementary table S2).

### Secondary Analysis—ICIQ‐OAB Scores

Within group improvements above minimum clinically important difference were observed for total ICIQ‐OAB and bother scores for active and sham stimulation between baseline and week 12 (see Fig. 1). Between active and sham stimulation, no significant difference was seen for ICIQ‐OAB sub‐components (see Supplementary Table S3). Assessing other time points, a significant difference was only seen at week 2 between arms, with a slightly worse score seen in the active arm (see Table S4), and no difference in findings was shown using a per‐protocol analysis.

### Secondary Analysis—Other Outcome Measures

No difference was seen between arms for bladder diary metrics at week 12, although strongly significant within group improvements in number of urgency episodes for both arms were observed between baseline and week 12 (see Supplementary Table S5). There was a moderately significant improvement in number of incontinence episodes recorded within group for active stimulation and modest improvement in nocturia for sham stimulation.

No difference was seen between arms when participants responded to a perceived global rating of change (including where stratified into “responder” groups—see Supplementary Section S3). No difference was seen between arms for the ICIQ male/female LUTS, ICIQ‐OAB quality of life, Scales for Outcomes in Parkinson's disease—Autonomic dysfunction (SCOPA‐AUT), or Patient Health Questionnaire 9 scores at week 12.

### Adverse Events

Adverse events occurring during the trial intervention period are described in Supplementary Table S6/Supplementary Section S4. Seventy‐six adverse events were recorded, of which 10 were deemed serious, although none attributed to the intervention. Leg pain was most common.

## Discussion

This study demonstrated no difference for both primary and secondary outcomes between active and sham arms, although both arms showed clinically significant within‐group differences.

Improved outcomes have been reported in many previous studies examining tibial nerve stimulation specifically for PD,^11^ although few have tested against a sham comparator.^10^, ^13^, ^14^ In two, superior outcomes were seen with active stimulation,^10^, ^13^ although the observed size of difference would not be classed as clinically meaningful. In a third study, statistical comparison was not made between the outcomes for patients between trial arms.^14^ The settings of the Arajuo and Perissinotto studies are not well described,^13^, ^14^ however, the current study was similar to STARTUP involving a United Kingdom (UK) population,^10^ albeit single as opposed to multi‐center.

A key consideration for this study is the intervention. Previous studies have used transcutaneous electrical nerve stimulation (TENS) machines with 10 to 20 Hz stimulation parameters, compared to the Geko device with a fixed frequency of 1 Hz. We selected the Geko device as an easy to use off the shelf solution that would be easier for a population with dexterity and potential cognitive issues to apply. The rationale was to improve concordance and negate the drawbacks of TENS requiring placement of multiple electrodes, settings, and the trip hazard of wires. One consideration is that the nature of Geko stimulation simply provides less benefit than the typical 10 to 20 Hz frequency used in TENS paradigms. With neuromodulation studies, there are near limitless variables that can be assessed (frequency, amplitude, duration, and interval), and it is important that the result of this study is considered only for 1 Hz TTNS with Geko.

The outcome of the trial could fit a placebo response, which is well described in continence research.^15^ Participants in the STRIPE spent more time focusing on symptoms and completing bladder diaries, which itself may have a temporary physical effect on symptoms.^16^ There is a recognized cognitive aspect to continence,^17^, ^18^ similar to other PD symptoms such as freezing of gait and tremor. This psychological mechanism is supported by the observation that although daytime symptoms improved, there was no change in nocturia. An alternative explanation to a potential placebo effect seen in STRIPE is that both arms are delivering a form of stimulation intervention, albeit with the markedly lower energy intervention in the sham arm trending toward superior. The development of sham/placebo interventions for any peripheral neuromodulation study is notoriously difficult.^19^ The sham paradigm chosen was a composite measure of removing device proximity from major nerve bundles (making motor stimulation near impossible) by moving the site of placement from medial to the lateral ankle, and differentiating between minimal and high levels of stimulation.

Strengths of this study include the use of a robust participant reported outcome measure relevant to OAB symptoms. We maintained high participant retention throughout the study.

We recognize several limitations. The achieved sample size was less than our power calculation, calculating 220 subjects would be required. This may have contributed to variations in Levodopa daily dose, fLUTS score, and nocturnal polyuria index at baseline between groups. Regardless, the 95% CI for the adjusted primary‐end point suggests that we can exclude a clinically meaningful benefit. Trial staff were not blinded to participant allocation because of practical limitations. However, this is mitigated by the primary outcome measure being determined by participants independently while still blinded, and if staff had introduced implicit bias, this did not translate to a beneficial primary outcome. Participants were given a single training session with no validation of correct placement/use during the trial duration undertaken except a single phone call at week 2 (resource limitations). It was also possible for participants to look‐up the true location of the tibial nerve, affecting blinding, and approximately one‐third of active participants were unable to achieve motor‐level stimulation.

Overall TTNS with the Geko was safe and generally well tolerated. The 10.2% overall drop‐out rate (including withdrawal/discontinuation) in STRIPE compares favorably with the small RCT by Perissinotto et al.^14^ and similar to the Araujo et al.^13^ and STARTUP studies (13.5% and 10.7%, respectively).^10^ Adverse events were not discussed in the Araujo and Perissinotto studies^13^, ^14^ (unclear if because of omission or the lack of any events), however, STARTUP actively stated there were no adverse events. Given the majority were because of pain, it is likely the 1 Hz as opposed to 10 Hz in STARTUP increased perception of intermittent muscle contraction and, therefore, pain.

## Conclusion

TTNS using the Geko device for OAB symptoms in PD does not appear to lead to any benefit over sham stimulation, and improvements above minimal clinically important difference were seen within both groups, suggesting a placebo effect, assuming that the sham paradigm had no biological effect. Although this phase II study was less powered than anticipated, the 95% confidence around our adjusted model suggests it is unlikely that active treatment would result in an important clinical benefit. This finding should not be generalized to other forms of TTNS because it is possible that different stimulation parameters might yield different effects and there were numerous study limitations. It is important to develop future studies, learning from the challenges inherit in this current trial when studying neuromodulation interventions for LUTS.

## Author Roles

MS conceived and designed the study, executed the study and analysis, and drafted the manuscript. GP/AC aided in study execution and analysis. MD, YBS, and EH aided in study design and drafting of the manuscript.

## Financial Disclosures

M.S. is funded by a Parkinson's UK Excellence Network Grant, has received honoraria for teaching provided on behalf of AbbVie and has received travel support from Medtronic. A.C. is funded by the University of Bristol. G.E.P. was supported by the Biotechnology and Biological Sciences Research Council‐funded South West Biosciences Doctoral Training Partnership. M.D. is funded by the National Institute for Health and Care Research (NIHR) and Rosetrees trust. Y.B.S. is Higher Education Funding Council for England (HEFCE) funded by University of Bristol and receives grant funding from the Wellcome Trust, Medical Research Council, Healthcare Quality Improvement Partnership, Parkinson's UK, Templeton Foundation, Versus Arthritis, Dunhill Medical Trust, National Institute for Health and Care Research, and the Gatsby Foundation; receives book royalties from Oxford University Press and Wiley books; consulting fees from Human Centric DD; is a member of the Trial Steering Committee for the SIMPLIFIED vitamin D RCT in chronic kidney disease patients; and is an unpaid member of the Alzheimer's Society grant board and Alzheimer's Research UK strategy committee. E.J.H. is HEFCE funded by University of Bristol for her academic work and has received research funding from the NIHR, the British Geriatrics Society, the Gatsby Foundation, the Alzheimer's Society, Royal Osteoporosis Society, the Dunhill Society, and Parkinson's UK. She has received travel support, honoraria, and/or sat on advisory boards for Kyowa Kirin, AbbVie, Luye, the CME institute, Ever, Simbec Orion, the Neurology Academy, and Bial.

## Supporting information


**Figure S1.** CONSORT diagram summarising flow within STRIPE trial. ITT, intention to treat; PP, per protocol.

**Table S1.** ICIQ OAB questionnaire scores for active and sham stimulation at week 12, with between arm comparison for active versus sham trial arm.
**Table S2.** Post‐hoc analysis stratifying primary outcome measure by sex, severity based on total ICIQ‐OAB score at baseline (≥11 or <11 derived from median score) and baseline urgency item severity (high severity = response of most of the time or all of the time), using linear regression model. Adjusted model uses baseline values as covariables.
**Table S3.** Scores for individual ICIQ OAB items at baseline and week 12, with post‐hoc analysis comparing difference between trial arms at week 12 using unadjusted and adjusted (including baseline scores as covariables) ordinal logistic regression model. NB: lower score indicates improved symptom.
**Table S4.** ICIQ OAB questionnaire main scores (A) and bother scores (B) at each two week assessment interval with linear regression models for active compared to sham stimulation arms. Adjusted model includes baseline ICIQ OAB total score as covariables. NB: lower score indicates improved symptoms *Remains significant after sensitivity analysis excluding influential outliers (*P* = 0.022).
**Table S5.** Within group differences in secondary outcome measures, and between group differences compared between arms at week 12 for secondary outcome measures, using ordinal logistic (OR) or linear regression (coefficient) models. Adjusted model uses baseline values as covariables.
**Table S6.** All adverse events occurring within STRIPE intervention period. MedDRA System Level and Organ Class designator highlighted in bold, lower level term for individual events below.

## Data Availability

The data that support the findings of this study are available from the corresponding author upon reasonable request.
