# Supplementary figures and images for: Transcutaneous Tibial Nerve Stimulation for Overactive Bladder Symptoms in Parkinson's Disease: Results from a Phase II Randomized Control Trial (STRIPE)
Source: Mov Disord. 2025 Apr 17;40(7):1291–6. doi: 10.1002/mds.30186 (PMC12273607; doi:10.1002/mds.30186)

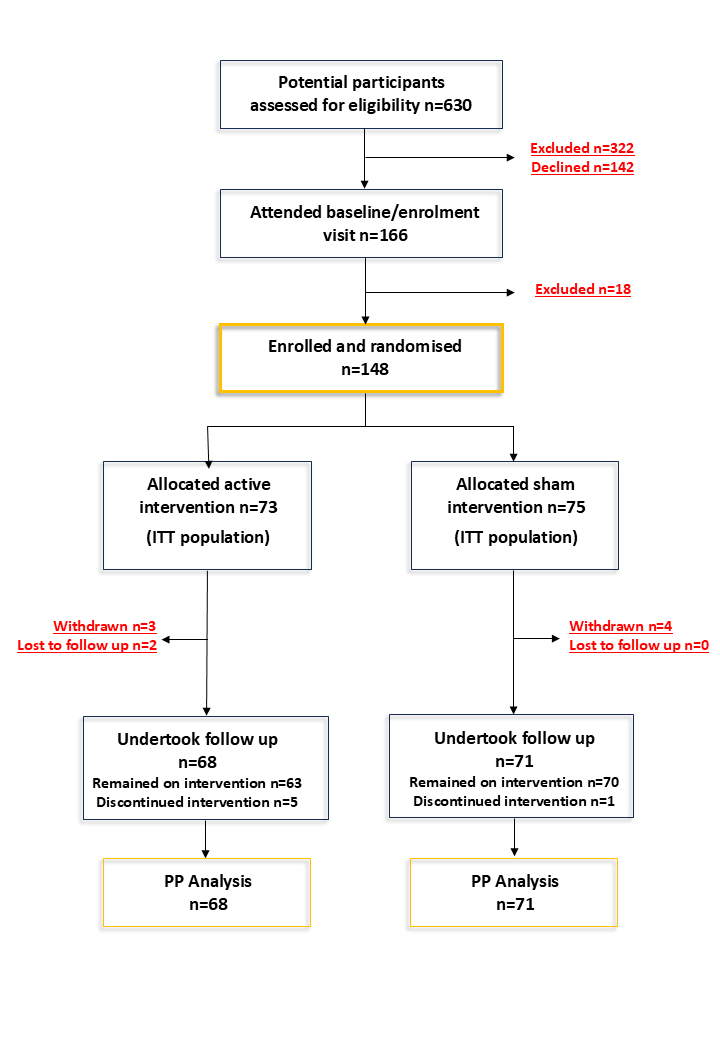

Supplement: Supplementary file 1 — Figure S1. CONSORT diagram summarising flow within STRIPE trial. ITT, intention to treat; PP, per protocol. [file MDS-40-1291-s002.png]
