# Supplementary material for: Transcutaneous Tibial Nerve Stimulation for Overactive Bladder Symptoms in Parkinson's Disease: Results from a Phase II Randomized Control Trial (STRIPE)
Source: Mov Disord. 2025 Apr 17;40(7):1291–6. doi: 10.1002/mds.30186 (PMC12273607; doi:10.1002/mds.30186)
Supplement: Supplementary file 2 — Table S1. ICIQ OAB questionnaire scores for active and sham stimulation at week 12, with between arm comparison for active versus sham trial arm. Table S2. Post‐hoc analysis stratifying primary outcome measure by sex, severity based on total ICIQ‐OAB score at baseline (≥11 or <11 derived from median score) and baseline urgency item severity (high severity = response of most of the time or all of the time), using linear regression model. Adjusted model uses baseline values as covariables. Table S3. Scores for individual ICIQ OAB items at baseline and week 12, with post‐hoc analysis comparing difference between trial arms at week 12 using unadjusted and adjusted (including baseline scores as covariables) ordinal logistic regression model. NB: lower score indicates improved symptom. Table S4. ICIQ OAB questionnaire main scores (A) and bother scores (B) at each two week assessment interval with linear regression models for active compared to sham stimulation arms. Adjusted model includes baseline ICIQ OAB total score as covariables. NB: lower score indicates improved symptoms *Remains significant after sensitivity analysis excluding influential outliers (P = 0.022). Table S5. Within group differences in secondary outcome measures, and between group differences compared between arms at week 12 for secondary outcome measures, using ordinal logistic (OR) or linear regression (coefficient) models. Adjusted model uses baseline values as covariables. Table S6. All adverse events occurring within STRIPE intervention period. MedDRA System Level and Organ Class designator highlighted in bold, lower level term for individual events below. [file MDS-40-1291-s001.docx]

**Supplementary data:**

***Section 1: methodology***

***Recruitment and enrolment***

Potential participants were identified through various methods: (i) clinician referral, (ii) self-referral following advertising by the Parkinson’s UK charity, (iii) subjects from the PRIME-UK cross-sectional study with relevant symptoms,^24^ and who had consented to be contacted about other research studies.

Participants attended a baseline assessment and enrolment session to participate, performed at the RICE centre in Bath, UK, where written consent was obtained. Information on basic demographics and clinical details such as medications and co-morbidities were recorded. Assessment with the Unified Parkinson’s Rating Scale (MDS-UPDRS) was performed. UTI was excluded on clinical grounds, although routine urinalysis was not performed. Inclusion criteria required participants to have a diagnosis of idiopathic PD (based on clinical diagnosis made by usual clinician) with a clinical history of OAB symptoms (urgency). Exclusion criteria are detailed in the protocol paper,^12^ however included implanted electronic device (DBS system, pacemaker). Information on severe structural genitourinary problems were discussed at screening as well as further assessed at baseline. Where severe active problems were present these patients were excluded**.** Similarly, patients with severe neuropathy were also excluded due to concern of the intervention being ineffective. Diabetes alone was not an exclusion criteria Participants could enrol if they had a history of nocturia alone, however were required to complete a screening 72-hour bladder diary to assess for the presence of severe nocturnal polyuria (nocturnal polyuria index >50%) which would lead to exclusion where no concurrent urgency history was reported. It is important to note that nocturia relates to the need to pass urine whilst sleeping, whilst nocturnal polyuria is one of many potential mechanisms of nocturia where excessive urine is produced at night, for example due to cardiac failure and is unlikely to respond to TTNS. Participants could enrol if they were taking bladder (anti-muscarinic, mirabegron) or prostate medication, however would have enrolment deferred if this had been started or the dose adjusted in the past three months. All potential participants were screened by telephone prior to attendance to prevent unnecessary attendance as far as possible.

Participants were asked to record any medication changes over the trial period, collected at follow up. Participants were unable to take part if they had changed a bladder medication (anti-muscarinic, mirabegron, prostate related agent) in the previous three months and were requested not to commence or change these during the trial duration. Where a change had been made in the three months before starting, enrolment was deferred.

***Randomisation and blinding***

1:1 randomisation was undertaken using Sealed Envelope (Sealed Envelope Ltd, London, UK). The minimization methodology is detailed in the procotol paper.^12^ Participants were blinded as to treatment allocation and the primary outcome was self-reported by them. It was impossible to blind the research team as they had to know whether the allocation was active or sham to supervise the correct placement of the TTNS device.

***Intervention***

Participants were allocated to either active or sham TTNS, undertaken as two 30-minute sessions per week, over the course of 12 weeks. Both arms used the Geko™ device (FirstKind Ltd, UK), currently licenced for use in venous thromboembolism prevention.^25^ This provides stimulation at 1hz with 11 adjustable pulse width settings. STRIPE was undertaken entirely independently from the manufacturer of the Geko™ device.

For active stimulation, participants were asked to place the device longitudinally behind the medial malleolus. Once placed, pulse width was increased until flexion or fanning of the toes was observed and continued at this level for the 30-minute session duration. For participants in whom this could not be achieved or tolerated, a secondary paradigm of maximum tolerated sensory stimulation was undertaken. For sham stimulation, participants were asked to place the device on the lateral malleolus. After placement, pulse width was left at the lowest setting for the duration of the session.

Participants were trained in device use during the baseline session, with the intention of them completing the 24 stimulation sessions at home. Support was available throughout the trial and formal follow up call to check compliance and fidelity of the intervention was undertaken at two weeks.

***Outcome measures***

The primary outcome measure was the International Consultation on Incontinence Questionnaire – Overactive bladder tool (ICIQ-OAB) at week 12.^26^ Participants were asked to complete ICIQ-OAB every two weeks in their stimulation diaries. Past research suggests that a one-point change is a minimum clinically important difference (MCID).^27^

Participants also completed the applicable ICIQ male or female LUTS questionnaire,^28,29^ ICIQ- Overactive Bladder Quality of Life module,^30^ and Scales for Outcomes in Parkinson’s disease- Autonomic questionnaires at baseline and at week 13, after completing the intervention.^12^

Participants were required to complete a 72-hour bladder diary prior to attending the initial enrolment visit as well as immediately after completing the intervention. The number of daytime and night voids, as well as frequency of urgency and incontinence episodes constituted further outcome measures.

A custom global rating of change score was created for STRIPE, related to the question “Compared to before you started, how much difference have you noticed after using the Geko devices for 12 weeks”. This was scored using a visual analogue scale rated from -5 (completely worse) to +5 (completely better).

***Safety assessment***

Participants were asked to contact the study team in the event of any perceived side effects beyond mild paraesthesia, or other significant health events such as hospitalization. Adverse events were assessed by the study team as to seriousness, severity and causality. An interim review was not undertaken, however adverse events were reviewed by an independent medical advisor at intervals throughout the trial.

***Statistical analysis***

Data was summarised using mean (with range and standard deviation) or median (with IQR) dependent on distribution or frequencies. Where distribution was found to be positively skewed, data was log_e_ transformed.

For primary outcome data, ICIQ-OAB scores between groups were assessed following 12 weeks of intervention after the treatment phase. Main ICIQ-OAB total score, and total bother score were analysed separately. Paired T-tests were undertaken to demonstrate within group differences. The primary and secondary analyses were undertaken using the principle of intention to treat (ITT), including all those randomised regardless of whether they received the intervention. A secondary per-protocol (PP) analysis was also undertaken.

For the primary outcome measure, an unadjusted and adjusted linear regression model was used comparing week 12 total ICIQ-OAB score between arms. Active stimulation was coded as 1 and sham stimulation was coded as 0, meaning the regression coefficients refer to the effect of exposure to active intervention versus sham. The adjusted model accounted for the baseline ICIQ-OAB score. Post-regression diagnostics were undertaken to assess the validity of the model, including the assessment of linearity and heteroscedasticity. Sensitivity analyses were performed, including the exclusion of influential outliers and multiple imputation with chained equations (MICE), to account for missing data points by pooling results from 150 imputed datasets. The 150 estimates are combined into an overall estimate and variance-covariance matrix using Rubin’s rules.^31^

Secondary outcomes were compared at week 13 (immediately post-treatment) between arms using a mixture of linear (bladder diaries, ICIQ mLUTS/fLUTS, SCOPA-AUT, PHQ9) and ordinal logistic (global rating of change) multivariable regression models used to test associations, unadjusted and adjusted for baseline responses.

Post-hoc analysis of individual ICIQ OAB score components and the effect of sex and severity of baseline symptoms were undertaken using further regression models. Statistical analysis used STATA version 18 (STATA corp LLC, USA) with data exported from a Redcap database (Vandebilt University, USA).

Sample size was calculated based on a single point difference being a minimum clinically important difference,^32^ using the standard deviation from a similar interventional study utilising ICIQ-OAB as a primary outcome measure.

***Section 2: Sensitivity and post-hoc analysis for primary outcome measure***

***Sensitivity analyses***

Controlling for factors that were unbalanced at baseline showed that there remained no difference at week 12 between trial arms for ICIQ OAB mean score (regression coefficient 0.85; CI -0.7, 2.4 P=0.26) and bother score (regression coefficient 2.2; CI -4.0, 8.4; P=0.48).

Due to the presence of missing data in the outcome variable for the primary model (with a missing rate of 13.5%), under the assumption of missing data being randomly distributed, we employed multiple imputation with chained equations (MICE) to impute the missing data. After imputation, there is no significant difference between the two arms, with a regression coefficient of 0.44 (95% CI: -0.3, 1.1; p = 0.21).

***Post-hoc analysis - change in individual ICIQ OAB components***

Changes in individual ICIQ OAB items at week 12 are displayed in supplementary table 3 as odd ratios for an increased (worse) score. No difference was seen for individual items between trial arms for frequency category or bother score.

***Post-hoc analysis – stratification by severity and sex***

No effect was seen when stratifying for participant sex (see supplementary table 2).

Participants were designated as having severe baseline symptoms if their initial ICIQ OAB score was ≥11 (the median score). Furthermore, participants were stratified based on baseline rating of urgency or urge incontinence, and designated as severe if their response was “most of the time” or “all of the time”.

Participants with severe total baseline scores in the active arm had a modestly significant higher ICIQ-OAB score at week 12 (unadjusted model: coefficient 0.85; %95 CI -0.02, 1.72; P=0.05, adjusted model: coefficient 1.02; %95 CI 0.28, 1.76, P=0.01). No difference between arms was seen for low severity baseline score, or where stratified by degree of urgency or urge incontinence (see supplementary table 2). There was no evidence of an interaction between trial arm and classification to high total score in the unadjusted model (P=0.44) or when adjusting for baseline (P=0.23).

***Section 3: Secondary outcome measures***

***Secondary analysis -Between group differences at other time points***

A significant difference between the arms was seen only at week 2 (see supplementary table 4), with a higher ICIQ OAB main score seen in the active arm within the adjusted model only (regression coefficient 0.69; 95% CI 0.2; 1.2; P=0.007). This remained significant after sensitivity analysis excluding influential outliers, however the association much weaker (regression coefficient 0.49; 95% CI 0.07; 0.9; P=0.022).

***Secondary analysis -Within group differences for ICIQ-OAB questionnaire***

Both active and sham arms demonstrated marked within-group differences for total ICIQ-OAB main score at week 12 compared to baseline (see figure 1A). These were greater than MCID value for the questionnaire (change in 1 point). The active arm improved by -1.2 points (95% CI -1.7, -0.6, P=0.001), whilst the sham arm improved by -1.7 points (95% CI -2.2, -1.3; P<0.001). Bother scores improved by -2.2 in the active arm (95% CI -4.3, -0.1; P=0.04) and -2.5 (95% CI-4.7, -0.4; P=0.02) in the sham arm (figure 1B).

***Secondary analysis – global rating of change***

Secondary outcome measures are summarised in supplementary table 5. No difference was seen between active and sham arms at week 12 for global rating of change (GRC) score (OR 1.08; CI 0.6, 2.0; P=0.80). A designation of “responder” was given to participants who reported a GRC ≥3 (range -5 to +5). 29 participants (21.2% of total) were classified as responders, 15 in the sham arm (21.3% of arm) and 14 in the active arm (21.2%). No difference in the odds of being a responder was seen between trial arms (OR 1.01; CI 0.4, 2.3; P=0.99). Secondary outcome variables are summarised in supplementary table 5.

***Secondary analysis – bladder diary outcomes***

No difference was seen between arms at week 12 (coefficient 0.32; 95% CI -0.4, 1.0; P=0.35 unadjusted, coefficient 0.22; 95% CI -0.4, 0.8; P=0.47 adjusted for baseline) for urgency episodes. Marked within subject improvements in the frequency of urgency episodes were seen for active (-1.3; 95% CI -1.9, -0.7; P<0.001) and sham (-1.1; 95% CI -1.6, -0.6; P<0.001) over baseline diaries.

No difference was seen between arms in the number of incontinence episodes at week 12 (coefficient 0.04 ; 95% CI -0.2, 0.3; P=0.75 unadjusted, coefficient -0.06; CI -0.3, 1.4; P=0.57 adjusted for baseline). Within subjects, a small improvement in the number of incontinence episodes was seen for active stimulation (coefficient: -0.2; 95% CI -0.4, -0.1, P=0.004) but not for sham stimulation (coefficient -0.1; 95% CI -0.3, 0.04; P=0.15), though the 95% confidence intervals overlap.

No difference was seen observed arms for the rate of nocturia at week 12 (coefficient -0.24; 95% CI; -0.6, 0.2; P=0.25 for unadjusted; coefficient -0.06; 95% CI -0.4, 0.2; P=0.68 for adjusted model based over baseline). No difference was seen for the within subject rate of nocturia over 12 weeks for active stimulation (coefficient -0.2; 95% CI-0.4, 0.04; P=0.11) and sham stimulation (coefficient -0.2; 95% CI -0.5, 0.03; P=0.08).

***Secondary analysis – additional questionnaires***

No difference was seen between trial arms at the week 13 point for any secondary outcome questionnaire. Small improvements were seen within groups for ICIQ OABqol score and SCOPA-AUT urinary sub-domain (supplementary table 5). Within group improvements in PHQ9 were seen for sham participants but not active participants. No within group differences were seen for overall SCOPA-AUT score.

***Section 4: Adverse events and safety***

Adverse events occurring during the trial intervention period are shown in supplementary table 6. 76 adverse events were recorded, of which 10 were deemed serious (being associated with hospital admission). All serious adverse events (SAE) were judged as not or unlikely related to the intervention. There was no association between intervention arm and the occurrence of an SAE (OR 1.03; 95% CI 0.3, 3.7; P=0.97). No participants died during the trial period following enrolment.

The most common adverse event was leg pain (n=23, 15.5% all participants), which was categorised as either nociceptive (n=19, 12.8% all participants) or neuropathic (n=3, 2.0% all participants). Pain was more common in the active compared to the sham arm (19 vs 3%, OR 9.0; 95% CI 2.6, 32.1 P<0.001). Next most common were urinary tract infections (n=13, affecting seven participants, 4.7%). No difference was seen between arms (OR 0.39; 95% CI 0.1, 2.1; P=0.28) Thereafter, skin irritation related to device placement was next most frequent (n=4, 2.7%), with no difference shown between arms (OR 0.33; 95% CI 0.1, 3.3; P=0.35).

Four participants discontinued due to adverse events attributed to the intervention; two due to gastrointestinal upset, one due to new onset of freezing and one due to leg swelling and erythema after falling asleep with the device *in situ* for one hour.

***Supplementary figure/table legends:***

**Supplementary figure 1:** **CONSORT diagram summarising flow within STRIPE trial. ITT=intention to treat, PP=per protocol.**

Supplementary table 1: ICIQ OAB questionnaire scores for active and sham stimulation at week 12, with between arm comparison for active versus sham trial arm

Supplementary table 2: Post-hoc analysis stratifying primary outcome measure by sex, severity based on total ICIQ-OAB score at baseline (≥11 or <11 derived from median score) and baseline urgency item severity (high severity = response of most of the time or all of the time), using linear regression model. Adjusted model uses baseline values as covariables.

**Supplementary table 3: Scores for individual ICIQ OAB items at baseline and week 12, with post-hoc analysis comparing difference between trial arms at week 12 using unadjusted and adjusted (including baseline scores as covariables) ordinal logistic regression model. NB: lower score indicates improved symptom**

Supplementary table 4: ICIQ OAB questionnaire main scores (A) and bother scores (B) at each two week assessment interval with linear regression models for active compared to sham stimulation arms. Adjusted model includes baseline ICIQ OAB total score as covariables. NB: lower score indicates improved symptoms *Remains significant after sensitivity analysis excluding influential outliers (p=0.022)

Supplementary table 5: Within group differences in secondary outcome measures, and between group differences compared between arms at week 12 for secondary outcome measures, using ordinal logistic (OR) or linear regression (coefficient) models. Adjusted model uses baseline values as covariables.

**Supplementary table 6: All adverse events occurring within STRIPE intervention period. MedDRA System Level and Organ Class designator highlighted in bold, lower level term for individual events below**

***Supplementary table 1:***

|  | **Active stimulation** | | | | **Sham stimulation** | | | | **Comparison of mean active week 12 scores vs sham** | | | | | |
| --- | --- | --- | --- | --- | --- | --- | --- | --- | --- | --- | --- | --- | --- | --- |
|  |  |  |  |  |  |  |  |  | ***Unadjusted model*** | | | ***Adjusted model*** | | |
|  | **Mean** | **Median** | **Range** | **SD** | **Mean** | **Median** | **Range** | **SD** | **Regression coefficient** | **95% confidence interval** | **P value** | **Regression coefficient** | **95% confidence interval** | **P value** |
| **ICIQ OAB main score** | 9.8 | 10 | 4-19 | 2.6 | 9.5 | 9 | 4-17 | 2.3 | 0.31 | -0.5, 1.1 | 0.45 | 0.48 | -0.2, 1.2 | 0.17 |
| **ICIQ OAB bother score** | 20.6 | 21 | 0-40 | 9.4 | 19.0 | 21 | 0-39 | 9.5 | 1.68 | -1.6, 5.0 | 0.31 | 1.78 | -1.2, 4.8 | 0.24 |

Supplementary table 1: ICIQ OAB questionnaire scores for active and sham stimulation at week 12, with between arm comparison for active versus sham trial arm

|  | **Active stimulation**  **(week 12)** | **Sham stimulation**  **(week 12)** | **Comparison of active week 12 scores vs sham** | | | | | |
| --- | --- | --- | --- | --- | --- | --- | --- | --- |
|  |  |  | ***Unadjusted model*** | | | ***Adjusted model*** | | |
|  |  |  | **Coefficient** | **95% CI** | **P Value** | **Coefficient** | **95% CI** | **P Value** |
| **A) ICIQ OAB main score** | | | | | | | | |
| **Male sex** | 9.8 | 9.2 | 0.59 | -0.44, 1.62 | 0.26 | 0.61 | -0.25, 1.47 | 0.16 |
| **Female sex** | 9.7 | 9.9 | -0.19 | -1.53, 1.15 | 0.78 | 0.24 | -0.88, 1.37 | 0.67 |
| **High baseline ICIQ-OAB score** | ***12.7*** | ***11.8*** | ***0.85*** | ***-0.02, 1.72*** | ***0.05*** | ***1.02*** | ***0.28, 1.76*** | ***0.01*** |
| **Low baseline ICIQ-OAB score** | 8.5 | 8.0 | 0.43 | -0.19, 1.06 | *0.18* | *0.46* | *-0.07, 1.01* | *0.09* |
| **High baseline urgency rating** | 11.3 | 9.9 | 0.20 | -1.95, 2.35 | 0.85 | 0.73 | -1.13, 2.58 | 0.44 |
| **Low baseline urgency rating** | 9.1 | 9.3 | -0.21 | -0.95, 0.54 | 0.58 | 0.01 | -0.64, 0.66 | 0.97 |
| **High baseline urge incontinence rating** | 4.0 | 4.2 | 1.94 | -0.51, 4.40 | 0.12 | 1.22 | -0.89, 3.33 | 0.25 |
| **Low baseline urge incontinence rating** | 2.5 | 2.4 | 0.21 | -0.63, 1.06 | 0.62 | 0.38 | -0.34, 1.1 | 0.30 |
| **B) ICIQ OAB bother score** | | | | | | | | |
| **Male sex** | 21.2 | 18.4 | 2.8 | -1.35, 6.96 | 0.18 | 1.41 | -2.10, 4.93 | 0.43 |
| **Female sex** | 19.7 | 20.0 | -0.23 | -5.65, 5.18 | 0.93 | -0.27 | -4.86, 4.32 | 0.91 |
| **High baseline ICIQ-OAB score** | 27.1 | 25.5 | 1.60 | -3.19, 6.39 | 0.51 | 1.95 | -2.31, 6.21 | 0.36 |
| **Low baseline ICIQ-OAB score** | 17.6 | 14.8 | 2.82 | -0.69, 6.34 | 0.11 | 1.31 | -1.81, 4.44 | 0.41 |
| **High baseline urgency rating** | 23.5 | 18.8 | 0.23 | 3.04, 3.50 | 0.89 | 1.96 | -6.78, 10.69 | 0.66 |
| **Low baseline urgency rating** | 19.3 | 19.0 | -1.84 | -11.7, 8.3 | 0.71 | -0.44 | -3.3, 2.4 | 0.76 |
| **High baseline urge incontinence rating** | 22.5 | 22.3 | 0.21 | -10.22, 10.64 | 0.97 | 0.06 | -8.72, 8.84 | 0.99 |
| **Low baseline urge incontinence rating** | 20.4 | 18.7 | 1.73 | -1.76, 5.21 | 0.33 | 0.81 | -2.15, 3.76 | 0.59 |

Supplementary table 2: Post-hoc analysis stratifying primary outcome measure by sex, severity based on total ICIQ-OAB score at baseline (≥11 or <11 derived from median score) and baseline urgency item severity (high severity = response of most of the time or all of the time), using linear regression model. Adjusted model uses baseline values as covariables.

|  | | **Active stimulation** | | **Sham stimulation** | | **Comparison of active week 12 scores vs sham** | | | | | |
| --- | --- | --- | --- | --- | --- | --- | --- | --- | --- | --- | --- |
|  |  |  |  |  |  | ***Unadjusted model*** | | | ***Adjusted model*** | | |
|  |  | **Baseline** | **Week 12** | **Baseline** | **Week 12** | **OR** | **95% CI** | **P Value** | **OR** | **95% CI** | **P Value** |
| **A) ICIQ OAB main score** | | | | | | | | | | | |
| **Day time Frequency** | **1-6 times**  **7-8 times**  **9-10 times**  **11-12 times**  **13 + times** | 31.5%  35.6%  26.0%  2.7%  4.1% | 35.8%  37.3%  20.9%  3.0%  3.0% | 28.4%  41.9%  21.6%  4.1%  4.1% | 40.9%  49.3%  5.6%  2.8%  1.4% | 1.69 | 0.9, 3.1 | 0.12 | 1.7 | 0.9, 3.3 | 0.13 |
| **Night time frequency** | **None**  **One**  **Two**  **Three**  **Four +** | 5.5%  23.3%  42.5%  20.6%  8.2% | 7.5%  38.8%  25.4%  20.9%  7.5% | 5.4%  17.6%  33.8%  20.3%  23.0% | 5.6%  32.4%  25.4%  18.3%  18.3% | 0.66 | 0.4, 1.2 | 0.17 | 1.27 | 0.6, 2.5 | 0.50 |
| **Urgency** | **Never**  **Occasionally**  **Sometimes**  **Most of the time**  **All of the time** | 1.4%  13.7%  54.8%  23.3%  6.9% | 4.5%  43.3%  32.8%  17.9%  1.5% | 0.0%  24.3%  47.3%  25.7%  2.7% | 5.6%  50.7%  36.6%  5.6%  1.4% | 1.60 | 0.9, 3.0 | 0.14 | 1.50 | 0.8, 2.9 | 0.22 |
| **Urge incontinence** | **Never**  **Occasionally**  **Sometimes**  **Most of the time**  **All of the time** | 9.6%  28.8%  52.1%  9.6%  0.0% | 16.4%  50.6%  23.9%  6.0%  3.0% | 8.1%  36.5%  41.9%  10.8%  2.7% | 21.1%  46.5%  29.6%  1.4%  1.4% | 1.21 | 0.6, 2.3 | 0.55 | 1.34 | 0.7, 2.6 | 0.39 |
| **B) ICIQ OAB bother score** | | | | | | | | | | | |
| **Day time Frequency** | **Mean bother** | 4.4 | 4.3 | 4.1 | 3.9 | 1.31 | 0.7, 2.4 | 0.38 | 1.20 | 0.6, 2.2 | 0.57 |
| **Night time frequency** |  | 5.0 | 4.6 | 5.1 | 4.5 | 1.07 | 0.6, 1.9 | 0.82 | 0.95 | 0.5, 1.7 | 0.85 |
| **Urgency** |  | 6.8 | 5.8 | 6.7 | 5.2 | 1.48 | 0.8, 2.7 | 0.19 | 1.25 | 0.7, 2.3 | 0.47 |
| **Urge incontinence** |  | 6.4 | 5.7 | 6.0 | 5.0 | 1.55 | 0.9, 2.8 | 0.14 | 1.23 | 0.7, 2.2 | 0.50 |

Supplementary table 3: Scores for individual ICIQ OAB items at baseline and week 12, with post-hoc analysis comparing difference between trial arms at week 12 using unadjusted and adjusted (including baseline scores as covariables) ordinal logistic regression model. NB: lower score indicates improved symptom

| **Time point** | **Active stimulation** | | | | **Sham stimulation** | | | | **Comparison of mean active week 12 scores vs sham** | | | | | |
| --- | --- | --- | --- | --- | --- | --- | --- | --- | --- | --- | --- | --- | --- | --- |
|  |  |  |  |  |  |  |  |  | ***Unadjusted model*** | | | ***Adjusted model*** | | |
|  | **Mean** | **Median** | **Range** | **SD** | **Mean** | **Median** | **Range** | **SD** | **Regression coefficient** | **95% confidence interval** | **P value** | **Regression coefficient** | **95% confidence interval** | **P value** |
| **A) ICIQ OAB main score** | | | | | | | | | | | | | | |
| **Week 2** | 10.9 | 11 | 7-16 | 2.0 | 10.4 | 11 | 5-17 | 2.1 | 0.52 | -0.2, 1.2 | 0.15 | ***0.69*** | ***0.2, 1.2*** | ***0.007**** |
| **Week 4** | 10.2 | 10 | 5-16 | 2.3 | 10.2 | 10 | 5-16 | 2.2 | 0.02 | -0.7, 0.8 | 0.95 | 0.11 | -0.5, 0.7 | 0.72 |
| **Week 6** | 10.0 | 10 | 5-17 | 2.5 | 10.0 | 9.5 | 5-18 | 2.4 | 0.04 | -0.8, 0.9 | 0.91 | 0.15 | -0.5, 0.8 | 0.66 |
| **Week 8** | 9.9 | 10 | 4-18 | 2.5 | 9.6 | 9 | 4-16 | 2.3 | 0.36 | -0.5, 1.2 | 0.39 | 0.38 | -0.3, 1.0 | 0.25 |
| **Week 10** | 9.7 | 10 | 4-16 | 2.4 | 9.6 | 9 | 4-15 | 2.2 | 0.11 | -0.7, 0.9 | 0.79 | 0.27 | -0.4, 0.9 | 0.42 |
| **Week 12** | 9.8 | 10 | 4-19 | 2.6 | 9.5 | 9 | 4-17 | 2.3 | 0.31 | -0.5, 1.1 | 0.45 | 0.48 | -0.2, 1.2 | 0.17 |
| **B) ICIQ OAB bother score** | | | | | | | | | | | | | | |
| **Week 2** | 23.8 | 25 | 4-40 | 7.9 | 22.3 | 24 | 4-40 | 8.5 | 1.06 | -1.7, 3.9 | 0.46 | 1.10 | -1.3, 3.5 | 0.37 |
| **Week 4** | 22.1 | 24 | 0-39 | 8.9 | 22.1 | 23 | 1-40 | 8.8 | -0.03 | -3.1, 3.0 | 0.98 | -1.7 | -2.9, 2.6 | 0.91 |
| **Week 6** | 20.9 | 21 | 0-39 | 9.2 | 21.1 | 22 | 0-40 | 9.4 | -0.20 | -3.4, 3.0 | 0.90 | -0.06 | -3.0, 2.9 | 0.97 |
| **Week 8** | 20.6 | 21 | 0-39 | 9.1 | 19.9 | 20 | 0-38 | 9.5 | 0.73 | -2.6, 4.0 | 0.67 | 0.27 | -2.7, 3.3 | 0.86 |
| **Week 10** | 21.2 | 22 | 0-39 | 9.1 | 19.5 | 21 | 0-37 | 9.7 | 1.67 | -1.7, 5.0 | 0.32 | 1.82 | -1.3, 5.0 | 0.25 |
| **Week 12** | 20.6 | 21 | 0-40 | 9.4 | 19.0 | 21 | 0-39 | 9.5 | 1.68 | -1.6, 5.0 | 0.31 | 1.78 | -1.2, 4.8 | 0.24 |

Supplementary table 4: ICIQ OAB questionnaire main scores (A) and bother scores (B) at each two week assessment interval with linear regression models for active compared to sham stimulation arms. Adjusted model includes baseline ICIQ OAB total score as covariables. NB: lower score indicates improved symptoms *Remains significant after sensitivity analysis excluding influential outliers (p=0.022)

| **Outcome measure** | **Active stimulation** | **Sham stimulation** | **Unadjusted model** | | | **Adjusted model** | | | |
| --- | --- | --- | --- | --- | --- | --- | --- | --- | --- |
|  |  |  | **OR** | **95% CI** | **P Value** | **OR** | **95% CI** | **P Value** | |
| Mean Global rating of change (GRC) | 1.1 | 1.2 | 1.08 | 0.6, 2.0 | 0.80 | n/a | | | |
| % responders  (GRC ≥3) | 21.2% | 21.1% | 1.01 | 0.4, 2.3 | 0.99 | n/a | | | |
|  | ***Within group difference*** | | ***Between group difference*** | | | | | | |
|  | **Active stimulation** | **Sham stimulation** | **Unadjusted model** | | | **Adjusted model** | | | |
|  |  |  | **Regression**  **coefficient** | **95% CI** | **P Value** | **Regression**  **coefficient** | **95% CI** | | **P Value** |
| Urgency episodes | **-1.3**  **(P<0.001)** | **-1.1**  **(P<0.001)** | 0.32 | -0.4, 1.0 | 0.35 | 0.22 | -0.4, 0.8 | | 0.47 |
| Incontinence episodes | **-0.2**  **(P=0.004)** | -0.1  (P=0.15) | 0.04 | -0.2, 0.3 | 0.75 | -0.06 | -0.3, 0.1 | | 0.57 |
| Nocturia episodes | -0.2  (P=0.11) | **-0.2**  **(p=0.08)** | -0.24 | -0.6, 0.2 | 0.25 | -0.06 | -0.4, 0.2 | | 0.68 |
|  | | | | | | | | | |
| ICIQ OAB qol score | **-12.5 (P<0.001)** | **-12.4 (P<0.001)** | -5.69 | -14.5, 3.1 | 0.20 | -2.25 | -9.8, 5.3 | | 0.56 |
|  |  |  |  |  |  |  |  | |  |
| SCOPA-AUT total | 0.1  (P=0.89) | -0.1 (P=0.87) | -0.47 | -3.5, 2.6 | 0.76 | 0.03 | -2.0, 2.1 | | 0.98 |
| SCOPA-AUT urinary | **-1.6 (P<0.001)** | **-1.5 (P<0.001)** | -0.48 | -1.7, 0.7 | 0.42 | -0.29 | -1.4, 0.8 | | 0.58 |
|  |  |  |  |  |  |  |  | |  |
| PHQ9 score | -0.8  (P=0.17) | **-1.36**  **(P=0.005)** | -0.05 | -1.7, 1.6 | 0.95 | 0.26 | -1.1, 1.6 | | 0.71 |

Supplementary table 5: Within group differences in secondary outcome measures, and between group differences compared between arms at week 12 for secondary outcome measures, using ordinal logistic (OR) or linear regression (coefficient) models. Adjusted model uses baseline values as covariables.

| **Event by system category** | **Active**  **n=73** | | **Sham**  **n=75** | |
| --- | --- | --- | --- | --- |
|  | Participants  (%) | Events | Participants  (%) | Events |
| **Cardiac disorders** | **1 (1.4)** | **1** | **1 (1.3)** | **1** |
| Palpitations | 1 (1.4) | 1 | 0 (0.0) | 0 |
| Ectopic heart beat | 0 (0.0) | 0 | 1 (1.3) | 1 |
| **Eye disorders** | **0 (0.0)** | **0** | 1 (1.3) | 1 |
| Retinal detachment | 0 (0.0) | 0 | 1 (1.3) | 1 |
| **Gastrointestinal disorders** | **2 (2.7)** | **2** | **2 (2.7)** | **2** |
| Constipation | 1 (1.4) | 1 | 1 (1.3) | 1 |
| Diarrhoea | 1 (1.4) | 1 | 0 (0.0) | 0 |
| Faecal incontinence | 0 (0.0) | 0 | 1 (1.3) | 1 |
| **General disorders and administration site conditions** | **1 (1.4)** | **1** | **0 (0.0)** | **0** |
| Swelling of toes | 1 (1.4) | 1 | 0 (0.0) | 0 |
| **Infections and infestations** | **3 (4.1)** | **9** | **6 (8.0)** | **6** |
| Cellulitis | 1 (1.4) | 1 | 0 (0.0) | 0 |
| Coronavirus infection | 0 (0.0) | 0 | 1 (1.3) | 1 |
| Urinary tract infection | 2 (2.7) | 8 | 5 (6.6) | 5 |
| **Injury, poisoning and procedural complications** | **2 (2.7)** | **2** | **0 (0.0)** | **0** |
| Head injury | 1 (1.4) | 1 | 0 (0.0) | 0 |
| Fall | 1 (1.4) | 1 | 0 (0.0) | 0 |
| **Musculoskeletal and connective tissue disorders** | **24 (32.9)** | **24** | **4 (5.3)** | **4** |
| Ankle sprain | 1 (1.4) | 1 | 0 (0.0) | 0 |
| Ankle swelling | 1 (1.4) | 1 | 0 (0.0) | 0 |
| Leg pain | 19 (26.0) | 19 | 1 (1.3) | 1 |
| Leg spasm | 1 (1.4) | 1 | 1 (1.3) | 1 |
| Neck pain | 0 (0.0) | 0 | 1 (1.3) | 1 |
| Pain in toe | 1 (1.4) | 1 | 0 (0.0) | 0 |
| Spinal osteoarthritis | 1 (1.4) | 1 | 0 (0.0) | 0 |
| Wrist fracture | 0 (0.0) | 0 | 1 (1.3) | 1 |
| **Neoplasms benign, malignant and unspecified** | **0 (0.0)** | **0** | **1 (1.3)** | **1** |
| Lung cancer | 0 (0.0) | 0 | 1 (1.3) | 1 |
| **Nervous system disorders** | **7 (9.6)** | **7** | **4 (5.3)** | **4** |
| Stroke | 1 (1.4) | 1 | 0 (0.0) | 0 |
| Neuropathic pain | 2 (2.7) | 2 | 1 (1.3) | 1 |
| Paraesthesia | 0 (0.0) | 0 | 1 (1.3) | 1 |
| Parkinson’s disease aggravated | 4 (4.1) | 3 | 1 (1.3) | 1 |
| Restless legs | 1 (1.4) | 1 | 1 (1.3) | 1 |
| **Psychiatric disorders** | **0 (0.0)** | **0** | **1 (1.3)** | **1** |
| Anxiety | 0 (0.0) | 0 | 1 (1.3) | 1 |
| **Renal and urinary disorders** | **1 (1.4)** | **1** | **0 (0.0)** | **0** |
| Urinary urgency | 1 (1.4) | 1 | 0 (0.0) | 0 |
| **Skin and subcutaneous tissue disorders** | **2 (2.7)** | **2** | **3 (4.0)** | **3** |
| Lipodermatosclerosis (bilateral) | 1 (1.4) | 1 | 0 (0.0) | 0 |
| Skin irritation | 1 (1.4) | 1 | 3 (4.0) | 3 |
| **Vascular disorders** | **1 (1.4)** | **1** | **2 (2.7)** | **2** |
| Postural hypotension | 1 (1.4) | 1 | 2 (2.7) | 2 |

***Supplementary table 6: All adverse events occurring within STRIPE intervention period. MedDRA System Level and Organ Class designator highlighted in bold, lower level term for individual events below***
